# Does Goal Attainment Scaling improve satisfaction regarding performance of activities of younger knee arthroplasty patients? Study protocol of the randomized controlled ACTION trial

**DOI:** 10.1186/s12891-016-0965-3

**Published:** 2016-03-02

**Authors:** Suzanne Witjes, Alexander Hoorntje, P. Paul F. M. Kuijer, Koen L. M. Koenraadt, Leendert Blankevoort, Gino M. M. J. Kerkhoffs, Rutger C. I. van Geenen

**Affiliations:** Department of Orthopaedic Surgery, Amphia Hospital, Molengracht 21, 4818 CK Breda, The Netherlands; Coronel Institute of Occupational Health, Academic Medical Center, Meibergdreef 9, 1100 DD Amsterdam, The Netherlands; Department of Orthopaedic Surgery, Academic Medical Center, Meibergdreef 9, 1100 DD Amsterdam, The Netherlands; ACES (Academic Centre for Evidence based Sports medicine), Amsterdam, The Netherlands; ACHSS (Amsterdam Collaboration for Health and Safety in Sports), AMC/VUmc, IOC Research Center, Amsterdam, The Netherlands

**Keywords:** Knee Osteoarthritis, Rehabilitation, Physiotherapy, Satisfaction, Activity, Goal attainment scaling, Randomized controlled trial

## Abstract

**Background:**

Knee arthroplasty is being increasingly performed, and also more often in a younger patient population (<65 years of age). Up to 20 % of patients remain dissatisfied after knee arthroplasty, despite the apparent technical success of the operation. Recent studies suggest that the fulfilment of patients’ expectations plays an important role in achieving satisfaction. Thus, addressing preoperative expectations more explicitly might improve patient satisfaction. The primary aim of the present study is to investigate the effect of a multidisciplinary, goal attained and individualized rehabilitation on satisfaction of activities of younger patients (<65 years) after knee arthroplasty.

**Methods/design:**

A single-centre randomized controlled trial will be conducted. In total, 120 patients (<65 years of age) with knee osteoarthritis who will undergo knee arthroplasty, will be randomly allocated to either goal attainment scaling rehabilitation or usual care rehabilitation. Goal attainment scaling rehabilitation includes drafting individually set rehabilitation goals preoperatively and measuring progress of rehabilitation on a six-point scale (-3 to +2). The primary outcome is patient satisfaction concerning activities in daily life, work and leisure time, including sports. Secondary outcome measures include KOOS, OKS, SQUASH and WORQ questionnaires and activity objectively measured with the Activ8® activity monitor.

**Discussion:**

The findings of this study will help to elucidate whether goal attainment scaling is an effective rehabilitation method for achieving higher levels of patient satisfaction, with a focus on activities, in younger patients after knee arthroplasty.

**Trial registration:**

This trial is since June 15^th^ 2015 registered at the Dutch Trial Register: NTR5251.

## Background

Osteoarthritis (OA) is a common chronic disease causing pain and disability among adults. In particular, the incidence of knee OA is high [1]. In the Netherlands, 594,000 persons suffer from knee OA [2]. Due to several external factors, such as aging of the population, epidemic obesity and secondary post-traumatic joint OA mainly caused by sports injuries, the prevalence of knee OA is expected to increase drastically in the Western world. At the same time, knee OA patients are becoming younger [3, 4].

Knee arthroplasty (KA), both total knee arthroplasty (TKA) and unicondylar knee arthroplasty (UKA), has proven to be a reliable form of treatment to relieve pain, improve function and enhance health-related quality of life [1]. Following the increasing prevalence of knee OA, the demand for KA is expected to rise worldwide [5]. In the Netherlands for example, an increase of 297 % of KAs from 2005 to 2030 is projected, with up to 57,900 KAs performed annually by 2030 [6]. Despite the aforementioned benefits of KA, 17–20 % of patients remain dissatisfied after surgery [7, 8]. In most of these cases, no implant-related mechanical failure can be found. The younger group of patients appears to expect to be more active and be able to perform more diverse activities after KA [3, 4]. These high preoperative expectations itself do not predict satisfaction after joint replacement [3], but *fulfilment* of these patient expectations clearly seems to play an important role in patient satisfaction [8, 9]. Current described percentages of fulfilment of expectations after KA range from 100 % satisfaction regarding knee pain alleviation to only about 20 % concerning the ability to participate in sports and leisure activities [8]. Although younger and more active patients may have higher expectations regarding activities, the expectations across patients vary highly for daily life, work and leisure time [10]. Patients expect to be able to return to work, but a recent study showed that the number of patients returning to work after KA is limited (<70 %) [11]. Concerning leisure time activities, a recent systematic review showed that return to intermediate- to high-impact sports is actually possible, and even more likely after UKA than after total knee arthroplasty (TKA) [12]. However, the literature suggests that patients often do not actually participate in functional levels of sports after KA [13]. Kersten et al. described that almost half of KA patients did not meet health-enhancing physical activity guidelines, and they were less active as a normative group [14].

Although the provision of postoperative physical therapy is almost universal, rehabilitation is the most understudied area concerning KA [15]. While short-term improvements of functional results with physiotherapy (PT) and exercise therapy after KA are shown in a recent systematic review, several aspects concerning rehabilitation after KA are unclear [16]. Recent surveys among physiotherapists showed that currently a great variety of PT methods is being used to attain patient goals in rehabilitation after KA [15, 17].

Considering the diverse expectations of this younger patient population on the one hand, and the importance of PT as part of rehabilitation on the other hand, the primary aim of the present study is to evaluate the effect of a multidisciplinary, goal attained and individualized rehabilitation on satisfaction of activities in daily life, work and leisure time including sports of younger patients (<65 years) after KA. The hypothesis is that multidisciplinary, goal attained and individualized rehabilitation, where patients’ goals are explicitly established and the feasibility of these goals is discussed and evaluated preoperatively, will result in higher patient satisfaction concerning these three domains compared to usual care physiotherapy rehabilitation.

## Methods/design

### CONSORT

In the description of our study design, we follow the Consolidated Standards of Reporting Trials (CONSORT statement) [18].

### Ethical principles

The study will be conducted according to the principles of the Declaration of Helsinki (59th World Medical Association General Assembly, Seoul, October 2008) and in accordance with the Medical Research Involving Human Subjects Act (WMO). Ethical approval has been received from the Medical Research Ethics Committee of the Academic Medical Center (AMC) Amsterdam. Patients will not be exposed to any potentially harmful procedures or therapies. No additional risks or discomfort are expected compared to standard rehabilitation. We only ask patients to fill in additional questionnaires preoperatively, after 3 months, after 6 months and after 1 year. This will take approximately 30 – 45 min. The online questionnaires can be completed at home at a convenient moment. In this way, we hope to minimize the extra burden for patients. There is no direct (financial) incentive for patients to participate in this trial. Patients’ motivation derives from the possible identification of a new treatment method that might benefit all future KA patients.

### Study design

**Fig. 1:**
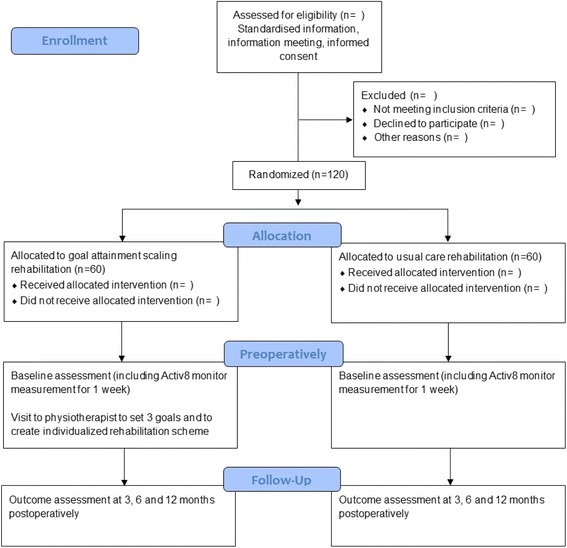
CONSORT Flow Diagram ACTION trial

The goAl attainment sCaling for knee arThroplasty In yOunger patieNts (ACTION) trial is a single-centre randomized controlled trial. Participants will be assigned to two groups: Usual care physiotherapy rehabilitation versus Goal Attainment Scaling (GAS) rehabilitation after KA. Figure 1 shows the CONSORT flow diagram of the ACTION trial.

### Study population

Patients that meet the following inclusion criteria are eligible for enrolment in this study:

- Patients < 65 years of age
- Debilitating knee osteoarthritis with an indication for KA, but not yet operated
- Currently participating in a job (either paid or voluntary)
- Able to perform usual rehabilitation care

If any of the following criteria apply, patients will be excluded:

- Cognitive or mental impairment
- No adequate levels of reading and writing the Dutch language
- Any disabling condition apart from knee osteoarthritis that restricts patients from performing their normal activities (e.g. pulmonary or cardiac disease, systemic inflammatory disease or pre-existing arthroplasty of hip or contralateral knee)

### Sample size

The primary outcome measure is a Visual Analogue Scale (VAS) for satisfaction regarding activities in daily life, work and leisure time, including sports. According to Singer et al. we use a minimal clinical relevant difference of ten points on a VAS scale from 0 to 100 with standard deviation of 15 [19]. Currently, data concerning how younger patients score satisfaction regarding activities in daily life, work and leisure time after KA are lacking. The best prediction can be extracted from the study of Kievit et al., in which young patients participating in a paid job with usual care after a KA scored a mean VAS score of 62 out of 100 points regarding work capacity [11]. According to these data, an intervention group has to score at least 72 (out of 100) points. Calculating with a power of 90 %, two-tailed testing with a *p*-value of 0.05, and standard deviation of 15 results in a minimum of 98 participants, 49 in the intervention group and 49 in the control group (nQuery Advisor® version 7.0). To adjust for 15 % dropouts over the 12 months follow-up period, 120 (60 per group) participants need to be included. It is expected that three patients per week can be included from the three locations of our hospital, so the inclusion period will cover approximately 40 weeks.

### Recruitment

All patients presenting to the orthopaedic outdoor clinics of the Amphia hospital in Breda, Oosterhout or Etten-Leur, who are indicated for KA (both TKA and UKA) and who meet inclusion criteria, will be invited to participate in the trial. One of the researchers (AH) will screen the KA waiting list for eligible patients and contact them to check if they are adequately informed and have received patient information. If this was not done initially, the patient will be informed and the patient information forms will be sent instead. If the patient has read the patient information and would like to participate, an appointment will be arranged for an inclusion meeting with the inclusion investigator (AH) on the same day the patient has to come to the hospital for another preoperative appointment (e.g. for visiting the anaesthesiologist or for a preoperative patient instruction meeting). During this inclusion meeting, the remaining questions of the potential participant can be answered. The participant will then sign the informed consent form, and after it has been signed by the investigator as well, the participant will receive a copy of it. The participants will be informed that their participation is voluntary and that they can withdraw from the study at any time. The patient will then be randomized to one of the two treatment arms and further instructions will directly follow in this inclusion meeting.

### Randomization and allocation concealment

Patients will be randomized in a 1:1 ratio to usual care physiotherapy rehabilitation or GAS rehabilitation. To conceal randomization, consecutively numbered, sealed, non-transparent envelopes will be prepared by one of the researchers (AH). Envelopes will be sorted in blocks of 10 for TKA patients and UKA patients separately. The envelopes will be stored in a locked location at the Amphia Molengracht. Randomization will take place during the inclusion meeting. By necessity, participants, researchers and physiotherapists are unblinded to group allocation.

### Intervention

**Fig. 2:**
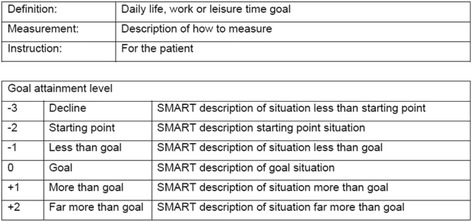
GAS scale

With patients wanting to participate in a great variety of activities postoperatively, standardized measurement tools do not always suffice in measuring what is relevant for the individual patient. GAS is a scoring method that measures the extent to which a patient’s individual goals are achieved by registering these goals on personal scales [20–22]. We will use the quality appraisal criteria for adequate scientific use of GAS scales that were recently suggested by Krasny-Pacini et al. [21] In our study, each goal is rated on a 6-point scale, ranging from -3 up to +2. The current situation is defined as -2 and the achievable goal for the patient after rehabilitation is marked as 0. A decline in performance is rated -3 and improvements are registered as -1, 0, +1 or +2. With GAS, the caregiver will create meaningful and relevant goals in collaboration with the patient. Secondly, these goals will be described at the International Classification of Functioning, Disability and Health (ICF) activity and participation level in accordance with the SMART (Specific, Measurable, Acceptable, Realistic and Time-specific) criteria [23]. After creating these individual goals, GAS scales have to be created by defining six realistic, distinct levels of outcome for -3, -2, -1, 0, +1 and +2, which will be used when evaluating progression during the rehabilitation. Patients in the intervention group preoperatively create their own rehabilitation goals, in collaboration with the selected and GAS-trained physiotherapist of their choice. Three goals will be set: one concerning a daily life activity, one concerning a work-related activity and one concerning a leisure time activity including sports which the patient wishes to perform better after KA. For these goals, GAS scales that can be used to objectify the progress in attaining these goals during follow-up will be created. Figure 2 shows an example of such a GAS scale.

The multidisciplinary research team will check if the GAS goals and scaling seem to be realistic for that individual patient, taking into account relevant baseline characteristics like age, BMI, comorbidities, preoperative physical activity level (SQUASH) and level of anxiety (HADS). When consensus concerning the rehabilitation goals and GAS scales has been reached, the treating physiotherapist will create an individualized treatment protocol for that individual patient, in accordance with the physiotherapists of the research team. The duration of the rehabilitation protocol may vary across patients but is estimated to be between 3 and 12 months. After KA surgery, standard in-hospital treatment and initial PT rehabilitation will be followed in both treatment groups. After hospital discharge, the patient will follow his/her personalized rehabilitation protocol.

### Usual care physiotherapy rehabilitation

Patients of the control group will follow usual care physiotherapy according to the expert opinion of their treating physiotherapists. Results of our survey among physiotherapists in the area of the Amphia hospital show that ≥95 % of therapists generally work on pain reduction and the improvement of range-of-motion, muscle strength, endurance and gait pattern (Witjes et al: Variable usual care rehabilitation and similar return to sports advice after knee arthroplasty: a survey amongst physiotherapists, submitted).

### Training of intervention physiotherapists performing GAS rehabilitation

Our multidisciplinary research team, consisting of two orthopaedic surgeons, two physiotherapists, a human movement scientist, a consultant for work-related musculoskeletal disorders and a researcher, followed an accredited course in GAS [24]. The research team will train the participating physiotherapists for the intervention group, which will be randomly selected (on a voluntary basis) from the group of physiotherapists that declared an interest in participating in the previous survey. Throughout the trial they will be guided and supervised by our multidisciplinary research team. To avoid selection bias, physiotherapists who treat patients from the intervention group will not treat patients from the control group and vice versa. In total, GAS-trained physiotherapists will be available at 25 physiotherapy locations where the intervention may take place. Another 30 locations have been selected where patients of the control group can follow usual care physiotherapy rehabilitation. Control group locations will also be randomly selected from the respondents to our previous survey.

### Data collection

Questionnaires will be collected digitally and stored on a password-protected, secured server to which only study staff will have access. The physiotherapists will send the GAS scales, rehabilitation schemes and GAS scores to the research team by email and these will be stored at a locked location.

### Outcome measures

Table 1 provides an overview of the outcome measures of this study and the follow-up moments.

**Table 1:** Overview of outcome measures collected at each time point

	Intervention group				Control group			
	Pre-operative	3 months	6 months	12 months	Preoperative	3 months	6 months	12 months
PROMs								
- VAS^a^	X	X	X	X	X	X	X	X
- KOOS	X	X	X	X	X	X	X	X
- OKS (-APQ)	X	X	X	X	X	X	X	X
- SQUASH	X	X	X	X	X	X	X	X
- WORQ	X	X	X	X	X	X	X	X
- HADS	X			X	X			X
- PAM	X			X	X			X
- NPS		X	X	X		X	X	X
- PT evaluation^b^		X	X	X		X	X	X
Performance measures								
- Activ8®	X		X		X		X	
- GAS scores	X	X	X					

^a^VAS concerning activities, primary outcome measure ^b^General questions on the delivery of physiotherapy

The primary outcome measure is a VAS for satisfaction concerning activities of daily life, work and leisure time including sports. The VAS is a validated, self-reported instrument that can accurately measure patient satisfaction on a scale ranging from 0 to 100, where zero is the worst score and 100 is the best score [19].

The secondary outcome measures consist of:

- The Knee injury and Osteoarthritis Outcome Score (KOOS) is a knee-specific questionnaire designed to assess symptoms and knee function in a young, physically active patient with knee damage and knee OA [25, 26]. It comprises five subscales (pain, other symptoms, function in daily living, function in sport and recreation, and knee-related quality of life) with a total of 42 questions. Standardized answering options are given (5-point Likert boxes) and each question is assigned a score from 0 to 4. A normalized score (100 indicating no symptoms and 0 indicating extreme symptoms) is calculated for each subscale.
- The Oxford Knee Score Activity and Participation Questionnaire supplement (OKS-APQ) is a supplement to the original OKS, developed to assess higher levels of activity and participation in younger and more active KA patients [27]. It contains eight extra questions, making 20 in all, on patients’ perspective concerning pain and function of the knee after KA [27–29]. Standardized answer options are given (5-point Likert boxes) and each question is assigned a score from 0 to 4. The sum of items results in a 0 – 48 score with 0 being the best score and 48 the worst.
- The Short QUestionnaire to ASsess Health-enhancing physical activity (SQUASH) is a self-reported questionnaire comprising questions on (A) daily living activities; (B) activities at work and school; (C) household activities; and (D) leisure time activities [30]. Questions concerning the frequency, duration and intensity of the performed activities are asked, and the number of minutes spent on each activity are totalled. A calculation can then be made to determine whether patients meet the Dutch Recommendation for Health-Enhancing Physical Activity (NNGB) [31]. In the Netherlands, the SQUASH is currently the standard instrument used to assess the physical activity level in the general Dutch population.
- The Work, Osteoarthritis or joint-Replacement Questionnaire (WORQ) was developed to assess physical difficulty experienced in work before or following TKA [32]. The questionnaire comprises 13 physical activities and patients are asked to grade the difficulty they have performing these activities on a five-point scale: none, mild, moderate, severe or extreme difficulty, corresponding with the scores 4, 3, 2, 1 and 0 respectively. The sum of the items is converted to a 0 – 100 score, with 100 being the best score and zero the worst.
- The Net Promotor Score (NPS) is a single question asking patients how likely it is that they would recommend their operation (KA) to friends or family [33]. It is a 0 – 10 point scale with zero representing very unlikely and ten very likely. Responses are divided into three groups: 10 – 9 are ‘promotors’, 8 – 7 are ‘passives’ and 6 – 0 are ‘detractors’. The overall score is then calculated by subtracting the percentage of detractors from the percentage of promotors, resulting in a score between -100 and +100.
- The Patient Activation Measure (PAM) is a non-knee-specific questionnaire for measuring the level of patient self-management concerning their health [34]. It contains 13 items and asks patients about their beliefs, knowledge and confidence concerning their engagement in a wide range of health behaviours. It assigns an activation score ranging from 0 to 100, based on the responses to the 13-item scale, with 100 representing the highest level of self-management.
- The Hospital Anxiety and Depression Scale (HADS) is a 14-item scale containing seven questions that relate to anxiety and seven questions that relate to depression [35, 36]. Each item is rated on a four-point scale ranging from 0 (not at all) to 3 (very often). Responses are based on the relative frequency of symptoms over the preceding week. Possible scores range from 0 to 21 for each subscale, with 0 meaning no symptoms and 21 meaning severe symptoms.
- Physiotherapy evaluation questions will be asked on topics such as the average duration and frequency of PT sessions, location of physiotherapy and patients’ satisfaction concerning their PT treatment.
- The Activ8® activity monitor is a newly designed, recently validated accelerometer that measures acceleration in three planes (3D) (Horemans et al, The Activ8 Activity Monitor: validation of detection of body postures and movements, submitted). It is a small device (2×3cm) that can be fixed with Tegaderm™ waterproof transparent dressing on the ventral side of the upper leg, halfway between hip and knee. This allows patients to take a shower while wearing the Activ8®. Patients will wear the Activ8® continuously for one week +/- 1 month preoperatively and continuously for one week 6 months postoperatively.
- GAS scores will be collected for patients in the intervention group at three and six months postoperatively.

### Data and statistical analysis

Analyses will be performed according to the intention-to-treat principle. In our power analyses, we accounted for 15 % loss to follow-up. Reasons for loss to follow-up will be explored. Patients’ baseline characteristics will be inspected to assess comparability between the two groups. Descriptive statistics will be presented for each group as the mean change (standard deviation, 95 % confidence intervals) in both primary and secondary outcomes from baseline to each time point, using IBM**®** SPPS software, version 19.0.

For our primary outcome measure, the VAS for activities, both mean postoperative values and mean differences between preoperative and postoperative values between intervention- and control groups will be compared. Our main outcome is the VAS score at twelve months postoperatively. Mixed-model statistics will be used to take into account the effects of time and treatment. Outcome will be presented as improvement of intervention group compared to control group, including 95 % confidence intervals. In the case of a positive treatment effect, additional information for reaching clinically relevant differences will be presented, such as number needed to treat (NNT) and absolute benefit increase (ABI).

For the analysis of the secondary outcomes KOOS, OKS – APQ, SQUASH, WORQ, NPS, PAM and HADS, both mean postoperative values (at diverse follow-up moments) and mean differences between preoperative and postoperative values between intervention- and control groups will be compared. Mann-Whitney U tests will be performed to compare total scores of these questionnaires between intervention- and control groups at the specified follow-up moments, namely at three, six and twelve months postoperatively.

Activ8® activity monitor data will be presented as the total amount of minutes per day spent on each activity. Differences in activity level between intervention- and control group, measured preoperatively and six months postoperatively, will be calculated using Mann-Whitney U tests. Finally, we will report on the GAS scores collected at three and six months postoperatively. GAS scores will be considered ordinal data and non-parametric analysis methods will be used. We will present the percentage of patients reaching their goals (0) as well as the percentage of patients reaching less than their goal (-1), more than their goal (+1) and far more than their goal (+2) [37].

## Discussion

This paper provides the study protocol of the ACTION trial, which is a single-centre RCT that will investigate the effect of individualized, goal attained rehabilitation on patient satisfaction for activities of daily life, work and leisure time including sports after KA. The main goal of this study is to establish whether GAS is an effective rehabilitation method for achieving higher levels of patient satisfaction compared to usual care physiotherapy rehabilitation. With up to 20 % of patients remaining dissatisfied after KA, it is important to find new ways to further improve satisfaction in this patient group. Rehabilitation after KA plays a crucial role in helping patients to regain functionality and thereby improve participation. Although first described in 1968, GAS has gained renewed attention in recent years. It has shown promising results in several paediatric rehabilitation and neurorehabilitation studies and its use in these practices is growing [21, 37]. However, its use in rehabilitation after orthopaedic surgery has never been studied. As orthopaedic patient populations become more diverse, the need for individualized outcome measurements, such as GAS, increases. With our study we hope to provide a preliminary insight into the usefulness of GAS in orthopaedic rehabilitation settings. The proposed 6-point scale, ranging from -3 to +2 (compared to the original 5-point scale ranging from -2 to +2), allows us to also define and measure a possible relapse in a patient’s performance (-3) [38]. Since GAS is described at the activity and participation level of the ICF, we believe that the GAS goals will be highly relevant to the patient. The ICF intends to provide a standard language and conceptual framework for the description of health-related states [39]. By using the ICF framework, involved health professionals can communicate more effectively, since the same description is used universally. In this way, the practice variation that was observed in previous physiotherapy studies might be reduced, which is desirable because of the increasing demand for effectiveness (including cost-effectiveness) in healthcare. Furthermore, goals will be described in compliance with SMART (Specific, Measurable, Acceptable, Realistic and Time-specific) criteria [23]. These SMART goals are both relevant to the patient and considered acceptable and realistic by the physiotherapist and a multidisciplinary team consisting of orthopaedic surgeons, physiotherapists, orthopaedic researchers and human movement scientists. We expect that using this method will increase the chances of actually reaching a certain goal, whereby patients’ expectations will be met more often and patient satisfaction will increase. Since we expect to find enough eligible patients in our centre to complete inclusion within 40 weeks, we have chosen a single-centre study setting.

The primary strength of the proposed study is its multidisciplinary approach, with orthopaedic surgeons, physiotherapists, orthopaedic researchers and human movement scientists all involved in the study. In other words, the effect of a multidisciplinary approach in the treatment of young, active knee arthroplasty patients will be investigated. The second strength of this study is the broad range of patient-reported outcome measures (PROMs) and performance-based measures (Activ8, GAS scores) that will be collected, thus representing actual patient satisfaction and activities in the best possible way. The outcome measures are all relatively easy to collect and our aim is to gain complete data sets. When using PROMs, using a 12-month follow-up is suggested since clinically significant improvements still occur in the six-to-twelve month recovery period [40]. Therefore, with a follow-up of 12 months and a considerable difference between our two treatment groups in terms of the rehabilitation program, we expect to find a relevant difference in both patient-specific and performance-based outcome measures.

Our aim with the ACTION trial is to investigate whether a multidisciplinary, individualized rehabilitation can aid orthopaedic surgeons and physiotherapists in achieving higher patient satisfaction levels in their younger (<65 years) KA patient population. Furthermore, a first impression on the practical use of GAS in daily physiotherapy practice for orthopaedic patients can be obtained.

## Abbreviations

GAS: goal attainment scaling
KA: knee arthroplasty
OA: osteoarthritis
TKA: total knee arthroplasty
UKA: unicondylar knee arthroplasty

## References

[1] Carr AJ, Robertsson O, Graves S, Price AJ, Arden NK, Judge A, Beard DJ. Knee replacement. Lancet. 2012;379:1331–40. 10.1016/S0140-6736(11)60752-6 22398175

[2] National Institute for Public Health and the Environment (2011). Nationaal Kompas Volksgezondheid [Dutch National Public Health Compass].

[3] Scott CEH, Bugler KE, Clement ND, MacDonald D, Howie CR, Biant LC (2012). Patient expectations of arthroplasty of the hip and knee. J Bone Jt Surg - Br Vol.

[4] Noble PC, Conditt MA, Cook KF, Mathis KB (2006). The John Insall Award: Patient expectations affect satisfaction with total knee arthroplasty. Clin Orthop Relat Res.

[5] Kurtz S, Ong K, Lau E, Mowat F, Halpern M (2007). Projections of primary and revision hip and knee arthroplasty in the United States from 2005 to 2030. J Bone Joint Surg Am.

[6] Otten R, van Roermund PM, Picavet HSJ (2010). Trends in the number of knee and hip arthroplasties: considerably more knee and hip prostheses due to osteoarthritis in 2030. Ned Tijdschr Geneeskd.

[7] Barlow T, Griffin D, Barlow D, Realpe A (2015). Patients’ decision making in total knee arthroplasty: a systematic review of qualitative research. Bone Jt Res.

[8] Bourne RB, Chesworth BM, Davis AM, Mahomed NN, Charron KDJ (2010). Patient satisfaction after total knee arthroplasty: Who is satisfied and who is not?. Clin Orthop Relat Res.

[9] Mancuso CA, Sculco TP, Wickiewicz TL, Jones EC, Robbins L, Warren RF, Williams-Russo P. Patients’ expectations of knee surgery. J Bone Joint Surg Am. 2001;83-A:1005–12. 10.2106/00004623-200107000-00005 11451969

[10] Witjes S, van Geenen RCI, Koenraadt KLM, van der Hart CP, Blankevoort L, Kerkhoffs GMMJ, Kuijer PPFM. Expectations of younger patients concerning activities after knee arthroplasty: Are we asking the right questions? Conditionnaly accepted for Qual Life Res. 10.1007/s11136-016-1380-9 PMC5288419 27492606

[11] Kievit AJ, van Geenen RCI, Kuijer PPFM, Pahlplatz TMJ, Blankevoort L, Schafroth MU (2014). Total knee arthroplasty and the unforeseen impact on return to work: a cross-sectional multicenter survey. J Arthroplasty.

[12] Witjes S, Gouttebarge V, Kuijer PPFM, Van Geenen RCI, Poolman RPW, Kerkhoffs GMMJ (2016). Return to Sports and Physical Activity After Total and Unicondylar Knee Arthroplasty: A Systematic Review and Meta-Analysis. Sport Med.

[13] Healy WL, Sharma S, Schwartz B, Iorio R (2008). Athletic activity after total joint arthroplasty. J Bone Joint Surg Am.

[14] Kersten RFMR, Stevens M, van Raay JJM, Bulstra SK, van den Akker-Scheek I (2012). Habitual Physical Activity After Total Knee Replacement. Phys Ther.

[15] Artz N, Dixon S, Wylde V, Beswick A, Blom A, Gooberman-Hill R (2013). Physiotherapy Provision Following Discharge after Total Hip and Total Knee Replacement: A Survey of Current Practice at High-Volume NHS Hospitals in England and Wales. Musculoskelet Care.

[16] Artz N, Elvers KT, Lowe CM, Sackley C, Jepson P, Beswick AD. Effectiveness of physiotherapy exercise following total knee replacement: systematic review and meta-analysis. BMC Musculoskelet Disord. 2015;16. 10.1186/s12891-015-0469-6 PMC4333167 25886975

[17] Naylor J, Harmer A, Fransen M, Crosbie J, Innes L (2006). Status of physiotherapy rehabilitation after total knee replacement in Australia. Physiother Res Int.

[18] Piaggio G, Elbourne DR, Altman DG, Pocock SJ, Evans SJW (2006). Reporting of noninferiority and equivalence randomized trials: an extension of the CONSORT statement. JAMA.

[19] Singer J, Thode HC (1998). Determination of the minimal clinically significant difference on a patient visual analog satisfaction scale. Acad Emerg Med.

[20] Kiresuk TJ, Sherman RE (1968). Goal attainment scaling: A general method for evaluating comprehensive community mental health programs. Community Ment Health J.

[21] Krasny-Pacini A, Hiebel J, Pauly F, Godon S, Chevignard M (2013). Goal Attainment Scaling in rehabilitation: A literature-based update. Ann Phys Rehabil Med.

[22] Turner-Stokes L (2009). Goal attainment scaling (GAS) in rehabilitation: a practical guide. Clin Rehabil.

[23] Bovend’Eerdt TJH, Botell RE, Wade DT (2009). Writing SMART rehabilitation goals and achieving goal attainment scaling: a practical guide. Clin Rehabil.

[24] Dekkers K, de Viet E, Eilander H, Steenbeek D (2011). Goal attainment scaling (GAS) in de praktijk [Goal attainment scaling in clinical practice].

[25] de Groot IB, Favejee MM, Reijman M, Verhaar JN, Terwee CB (2008). The Dutch version of the Knee Injury and Osteoarthritis Outcome Score: a validation study. Health Qual Life Outcomes.

[26] Roos EM, Lohmander LS (2003). The Knee injury and Osteoarthritis Outcome Score (KOOS): from joint injury to osteoarthritis. Health Qual Life Outcomes.

[27] Dawson J, Beard DJ, McKibbin H, Harris K, Jenkinson C, Price J (2014). Development of a patient-reported outcome measure of activity and participation (the OKSAPQ) to supplement the Oxford knee score. Bone Jt J.

[28] Haverkamp D, Breugem SJM, Sierevelt IN, Blankevoort L, van Dijk CN (2005). Translation and validation of the Dutch version of the Oxford 12-item knee questionnaire for knee arthroplasty. Acta Orthop.

[29] Murray DW, Fitzpatrick R, Rogers K, Pandit H, Beard DJ, Carr AJ, Dawson J. The use of the Oxford hip and knee scores. J Bone Jt Surg. 2007;89:1010–4. 10.1302/0301-620X.89B8.19424 17785736

[30] Wendel-Vos GCW, Schuit J, Saris WHM, Kromhout D (2003). Reproducibility and relative validity of the short questionnaire to assess health-enhancing physical activity. J Clin Epidemiol.

[31] Kemper H, Ooijendijk W (2000). Consensus over de Nederlandse norm voor gezond bewegen [Consensus on the Dutch Healthy Exercise Norm]. Tijdschr voor gezondheidswetenschappen.

[32] Kievit AJ, Kuijer PPFM, Kievit R, Sierevelt IN, Blankevoort L, Frings-Dresen MHW (2014). A Reliable, Valid and Responsive Questionnaire to Score the Impact of Knee Complaints on Work Following Total Knee Arthroplasty: The WORQ. J Arthroplasty.

[33] Hamilton DF, Lane JV, Gaston P, Patton JT, MacDonald DJ, Simpson HRW, Howie CR. Assessing treatment outcomes using a single question: The Net Promoter Score. Bone Jt J. 2014;96(B):622–8. 10.1302/0301-620X.96B5.32434 24788496

[34] Hibbard JH, Stockard J, Mahoney ER, Tusler M (2004). Development of the Patient Activation Measure (PAM): conceptualizing and measuring activation in patients and consumers. Health Serv Res.

[35] Bjelland I, Dahl AA, Haug TT, Neckelmann D (2002). The validity of the Hospital Anxiety and Depression Scale. J Psychosom Res.

[36] Blackburn J, Qureshi A, Amirfeyz R, Bannister G (2012). Does preoperative anxiety and depression predict satisfaction after total knee replacement?. Knee.

[37] Steenbeek D, Ketelaar M, Galama K, Gorter JW (2007). Goal attainment scaling in paediatric rehabilitation: A critical review of the literature. Dev Med Child Neurol.

[38] Steenbeek D, Meester-Delver A, Becher JG, Lankhorst GJ (2005). The effect of botulinum toxin type A treatment of the lower extremity on the level of functional abilities in children with cerebral palsy: evaluation with goal attainment scaling. Clin Rehabil.

[39] Kostanjsek N (2011). Use of The International Classification of Functioning, Disability and Health (ICF) as a conceptual framework and common language for disability statistics and health information systems. BMC Public Health.

[40] Browne JP, Bastaki H, Dawson J (2013). What is the optimal time point to assess patient-reported recovery after hip and knee replacement? A systematic review and analysis of routinely reported outcome data from the English patient-reported outcome measures programme. Health Qual Life Outcomes.

